# Validity and reliability of the Japanese versions of the coronavirus anxiety scale for adolescents and obsession with COVID-19 scale for adolescents

**DOI:** 10.7717/peerj.15710

**Published:** 2023-08-09

**Authors:** Takuya Makino, Sohei Ide, Tomoko Shiino, Daiki Hiraoka, Saeko Ishibashi, Futoshi Suzuki, Shota Nishitani

**Affiliations:** 1 Research Center for Child Mental Development, University of Fukui, Eiheiji, Fukui, Japan; 2 Department of Child and Adolescent Psychological Medicine, University of Fukui Hospital, Eiheiji, Fukui, Japan; 3 Division of Developmental Higher Brain Functions, United Graduate School of Child Development, Osaka University, Kanazawa University, Hamamatsu University School of Medicine, Chiba University, and University of Fukui, Suita, Osaka, Japan; 4 Department of Neuropsychiatry Faculty of Medical Sciences, University of Fukui, Eiheiji, Fukui, Japan; 5 New Institute of SocioInfonomics, Tama University, Meguro, Tokyo, Japan; 6 Center for Education in Liberal Arts and Sciences, Osaka University, Suita, Osaka, Japan; 7 Division of Developmental Neuroscience, United Graduate School of Child Development, Osaka University, Kanazawa University, Hamamatsu University School of Medicine, Chiba University, and University of Fukui, Suita, Osaka, Japan; 8 Department of Psychology, Faculty of Literature, Kobe Shinwa University, Kobe, Hyogo, Japan; 9 Japan Society for the Promotion of Science, Chiyoda, Tokyo, Japan; 10 Graduate School of Medical Sciences, University of Fukui, Eiheiji, Fukui, Japan; 11 Faculty of Nursing Science, Tsuruga Nursing University, Tsuruga, Fukui, Japan; 12 Department of Child and Adolescent Psychological Medicine, University of Fukui Hospital, Eiheiji, Fukui, Japan; 13 Life Science Innovation Center, University of Fukui, Fukui, Fukui, Japan

**Keywords:** Anxiety, COVID-19, Adolescent, Obsessive thought, Questionnaire, Japanese, Reliability, Validity

## Abstract

**Background:**

The coronavirus disease 2019 (COVID-19) has caused mental health issues in both adults and adolescents. The Coronavirus Anxiety Scale (CAS) and Obsession with COVID-19 Scale (OCS) questionnaires measure anxiety and persistent and disturbed thoughts (also known as obsessions) related to COVID-19. We developed Japanese versions of the CAS (*i.e.*, CAS-JA) and OCS (*i.e.*, OCS-JA) questionnaires to make them suitable for adolescents and validated the characteristics of these scales.

**Methods:**

Two online surveys were administered to high school students aged 15–18 years. A total of 263 students participated in the first survey and almost half of them participated in the second survey. In the first survey, participants responded to the CAS-JA, OCS-JA, generalized anxiety and obsessive–compulsive subscales of the Spence Children’s Anxiety Scale (SCAS), and Kessler 6 Scale (K6). The SCAS and K6 were used to verify discriminant validity and inter-scale correlations. In the second survey, the participants completed the CAS-JA and OCS-JA again to verify test–retest reliability. We performed a confirmatory factor analysis and calculated the model fit indices. Additionally, we examined the internal consistency reliability, convergent validity, and inter-item correlations of the CAS-JA and OCS-JA. Moreover, differences in CAS-JA and OCS-JA responses by gender and region of residence (state of emergency and non-emergency areas) were examined.

**Results:**

The results of the single-factor model confirmatory factor analysis of model fit indices were above the threshold. The required criteria for internal consistency reliability, test–retest reliability, and discriminant and convergent validity were met in both the CAS-JA and OCS-JA. No statistically significant differences attributed to residence and gender were found in both questionnaires.

**Conclusions:**

The results indicate that the CAS-JA and OCS-JA questionnaires are useful in measuring COVID-19-related anxiety, and persistent and disturbed thoughts in Japanese adolescents.

## Introduction

The coronavirus disease 2019 (COVID-19) pandemic has persisted worldwide and caused unprecedented losses. By May 22, 2022, 8,583,048 people had been infected, and 30,287 deaths were reported in Japan (Dong, Du & Gardner, 2020; Johns Hopkins University of Medicine, 2021). The Japanese government closed schools, restricted outdoor activities, and finally declared a national state of emergency in April 2020. Since the spread of COVID-19, schools have cancelled regular classes and reduced events. Leisure facilities also closed, or opened for business with shortened hours of operation, and restricted entry, thus reducing opportunities for families and peers to spend outdoor time together. In adjusting to such unprecedented changes, children and adolescents often display behavioral changes and tend to have emotional issues. A systematic review showed that the COVID-19 pandemic has caused children and adolescents to experience mental health issues such as anxiety, depression, irritability, boredom, sleep disturbances, excessive fear, and inattention (Panda et al., 2021). Moreover, exacerbation of symptoms in children and adolescents with obsessive-compulsive disorder during the COVID-19 pandemic has been reported in Turkey (Tanir et al., 2020). A Spanish study has also demonstrated psychological and behavioral changes in children and adolescents during this period. Symptoms such as anxiety, depression, problems with emotional regulation, and physical complaints are reportedly higher among adolescents than among young children (Pizarro-Ruiz & Ordóñez Camblor, 2021).

In light of the above, a simple screening tool is urgently needed for assessing COVID-19-related anxiety, and persistent and disturbed thoughts (also known as obsessions) in adolescents, to address their mental health issues associated with COVID-19. Recently, two psychological questionnaires: the Coronavirus Anxiety Scale (CAS) and Obsession with COVID-19 Scale (OCS), were developed by Lee to measure these COVID-19-related anxiety, and persistent and disturbed thoughts (Lee, 2020a; Lee, 2020b). These questionnaires are simple Likert scales that have been translated into many languages (Magano et al., 2021; Ahmed et al., 2022; Ashraf, Lee & Elizabeth Crunk, 2022; Broche-Pérez et al., 2022; Caycho-Rodríguez et al., 2022; Choi, Lee & Lee, 2022; Evren et al., 2022). The Japanese versions of these scales (CAS-J/OCS-J) have been developed and their psychometric properties validated (Ishibashi et al., 2022). However, because the original questionnaires and their Japanese versions (CAS-J/OCS-J) were not explicitly developed for adolescents, they can be difficult to understand and answer. Anxiety disorders and obsessive–compulsive disorders sometimes begin at a young age (Viana & Andrade, 2012), making it crucial to capture these COVID-19-related symptoms accurately.

However, certain items on the CAS-J and OCS-J questionnaires might not be easy for adolescents to understand because the scales use a few characters that are difficult to comprehend. The Japanese language uses combinations of three forms of characters, namely, “*hiragana*,” “*katakana*,” and “*kanji*” (Chinese characters), the last being particularly difficult. The adult version of the questionnaires contains passages that use kanji, which middle and high schoolers may be unable to comprehend. Considering that these questionnaires will be administered to adolescents with neurodevelopmental disorders, pathological anxiety, obsessions, and low cognitive functioning, we believe that it is necessary to create a version adolescents can easily respond to. We therefore revised the questions and answer choices, limiting the expressions and the usage of kanji to the knowledge level of a typical middle-school student in Japan.

Although adolescents showed low susceptibility to the alpha variant, they have shown high susceptibility to the recently spreading Omicron variant. Therefore, this study developed Japanese adolescent versions of the CAS and OCS and validated their psychometric properties.

## Materials & Methods

### Participants

The participants were recruited through Rakuten Insight, Inc., a Japanese online survey research company. We designed two online surveys for high school students aged between 15 and 18 years. Data cleaning was performed, and a first and second batch of 263 and 130 participants, respectively, was included in the analysis. The second survey was conducted to assess test–retest reliability. Participants’ sociodemographic characteristics are listed in Table 1. The first survey was conducted on March 3, 2021, and the second on March 10–11, 2021. The participants were provided incentives by the research company.

**Table 1:** Sociodemographic characteristics.

*n* = 263		*n*	(%)
Gender	Male	62	(23.6)
	Female	201	(76.4)
Age (years)	15	4	(1.5)
	16	63	(24.0)
	17	85	(32.3)
	18	111	(42.2)
Region	State of emergency area	89	(33.8)
	Other area	174	(66.2)

### Ethics

The study protocol and procedures were approved by the Research Ethics Committee of the University of Fukui (Assurance 2020177) and performed in accordance with the Declaration of Helsinki guidelines. Prior to filling in the questionnaires, participants were presented with a screen explaining the study. They were informed that participation was voluntary, parental permission was required for participation, and answering the questionnaire constituted consent.

### Measures

The CAS questionnaire evaluates COVID-19 anxiety (Lee, 2020a). It is a five-item, five-point Likert scale (0–4) (Broche-Pérez et al., 2022). The OCS questionnaire evaluates persistent and disturbed thoughts (also known as obsessions) related to COVID-19 (Lee, 2020b; Ashraf, Lee & Elizabeth Crunk, 2022). It is a four-item, five-point Likert scale (0–4), wherein higher scores indicate greater persistent and disturbed thoughts associated with COVID-19 (Ashraf, Lee & Elizabeth Crunk, 2022). Permission from the original authors of the CAS and OCS was obtained, and the questionnaire was translated into Japanese by two licensed psychologists (TM and TS) to make it suitable for adolescents. To verify the accuracy of the translation, a back translation by a translation company was done to compare it with the original version. The accuracy of the translation was verified, and the CAS-JA and OCS-JA were created.

To assess the discriminant validity and inter-scale correlations of the CAS-JA and OCS-JA, the Japanese versions of the Spence Children’s Anxiety Scale (SCAS) and the Kessler 6 Scale (K6) were administered. The SCAS questionnaire measures anxiety disorders and obsessive-compulsive disorder symptoms in children (Spence, 1998; Ishikawa et al., 2018). It consists of 38 items across six subscales, with responses rated on a four-point Likert scale (0 −3). Among these items, six items are related to generalized anxiety disorder (SCAS-GAD) and six to obsessive-compulsive disorder (SCAS-OCD). This study used these 12 items. The K6 questionnaire measures psychological distress (Kessler et al., 2003; Furukawa et al., 2008), with six items rated on a five-point Likert scale (0–4) and scores ranging from 0–24. The authors received permission to use the CAS, OCS, and SCAS from the copyright holders. The K6 could be used without permission. Certain terms used on the scales were revised and changed before the web survey. We could not technically use the 0 option in the survey; therefore, we modified all scales to start at 1 instead. During data analysis, responses were processed according to the original scaling of the questionnaire.

### Data analysis

We conducted confirmatory factor analysis (CFA) to verify if the CAS-JA and OCS-JA had single-factor structures. We used the weighted least squares mean and variance adjusted method (WLSMV) for estimation. The WLSMV method uses diagonal weighted least squares as an estimator, a robust standard error, and a scaled and shifted test statistic (Asparouhov & Muthén, 2010; Shipley, 2016). We checked the chi-squared/df value, comparative fit index (CFI), Tucker Lewis index (TLI), root mean square error of approximation (RMSEA), and standardized root-mean-square residual (SRMR). Ordinal omega and ordinal alpha indices were calculated for internal consistency reliability. We conducted a second survey with the original participants for test–retest reliability and calculated the intraclass correlation coefficients (ICC) for evaluation. To examine the discriminant validity between the scales, we calculated the correlation coefficient and heterotrait-monotrait ratio of correlation (HTMT) between the scales. Based on the Fornell & Larcker (1981) criterion, composite reliability (CR) and average variance extracted (AVE) were calculated to show the convergent validity of the CAS-JA and OCS-JA within scales. Inter-item correlations were also evaluated. First, the Shapiro–Wilk test was used to assess the normality of all questionnaires and their items, which could not be confirmed. Therefore, we decided to use Spearman’s rank correlations. We assessed whether the region of residence (state of emergency and non-emergency areas declared by the government) and the gender of the participants confounded the total scores of the CAS-JA and OCS-JA. We also examined whether regional status or gender had a statistically significant effect on CAS-JA and OCS-JA, using the Wilcoxon rank-sum test (WRS test) and the CFA model for these comparisons. Analyses were conducted using R (version 4.1.3), with the packages lavaan (version 0.6-11), semTools (version 0.5-5), psych (version 2.2.3), irr (version 0.84.1), and Mplus software (version 8.7).

## Results

### Descriptive statistics of the CAS-JA and OCS-JA

The response rates, means, and standard deviations for the CAS-JA and OCS-JA are shown in Tables 2 and 3, respectively. Most responses on the CAS-JA and OCS-JA were zero, indicating that anxiety and persistent and disturbed thoughts related to COVID-19 were not high among Japanese high school students at the time of measurement.

**Table 2:** Psychometric properties of CAS-JA.

	Response scale					Descriptive		Spearman’s rho			
	0	1	2	3	4	Mean	SD	2	3	4	5
Item 1	85.932	4.563	4.943	1.901	2.662	0.308	0.865	0.515^***^	0.655^***^	0.504^***^	0.522^***^
Item 2	87.452	7.985	2.662	1.141	0.760	0.198	0.611		0.530^***^	0.521^***^	0.521^***^
Item 3	92.776	2.281	1.901	2.281	0.760	0.160	0.634			0.607^***^	0.648^***^
Item 4	92.395	3.422	1.521	2.281	0.380	0.148	0.584				0.619^***^
Item 5	94.297	2.281	1.521	1.901	0.000	0.110	0.493				

**Notes.**

SD, standard deviation.

*** *p* < 0.001.

### Factor structures

Standardized factor loadings for each item on the CAS-JA ranged from 0.829 to 0.956, and those on the OCS-JA ranged from 0.704 to 0.918. The model fit indices for the CAS-JA were chi-squared/ *df* = 5.322/5 = 1.064, CFI = 1.000, TLI = 1.000, RMSEA = 0.0016, and SRMR = 0.015, and those of the OCS-JA were chi-squared/df value = 1.691/2 = 0.846, CFI = 1.000, TLI = 1.000, RMSEA = 0.000, and SRMR = 0.012. The thresholds of CFI and TLI were proposed to be >0.90, RMSEA <0.10, and SRMR <0.05. All indices met or exceeded the recommended thresholds.

**Table 3:** Psychometric properties of OCS-JA.

	Response scale					Descriptive		Spearman’s rho		
	0	1	2	3	4	Mean	SD	2	3	4
Item 1	70.722	20.913	4.563	3.042	0.760	0.422	0.782	0.668^***^	0.639^***^	0.377^***^
Item 2	77.186	15.589	4.183	2.281	0.760	0.338	0.733		0.581^***^	0.421^***^
Item 3	75.665	12.928	6.464	1.901	3.042	0.437	0.926			0.441^***^
Item 4	88.593	7.224	3.422	0.760	0.000	0.163	0.502			

**Notes.**

SD, standard deviation.

*** *p* < 0.001.

### Internal consistency reliability

Ordinal omega values for the CAS-JA and OCS-JA were 0.97 and 0.92, respectively. Ordinal alphas for the CAS-JA and OCS-JA were 0.95 and 0.91, respectively. The threshold for internal consistency reliability using ordinal omega and ordinal alpha was more than 0.7 (Cortina, 1993); thus, the CAS-JA and OCS-JA met the requirement for internal consistency.

### Test–retest reliability

The ICC for the CAS-JA and OCS-JA were 0.839 and 0.804, respectively. Cicchetti (1994) suggested that an ICC value of 0.75 or higher has excellent reliability. Hence, the test–retest reliability of the CAS-JA and OCS-JA met the requirement.

### Discriminant validity and inter-scale correlations

We calculated the correlation coefficient between the total scores of each scale; that of the CAS-JA and SCAS-GAD was 0.328 (*p* < 0.001); that of the CAS-JA and K6 was 0.355 (*p* < 0.001); that of the OCS-JA and SCAS-OCD was 0.460 (*p* < 0.001), and that of the OCS-JA and K6 was 0.399 (*p* < 0.001).

HTMT for the CAS-JA and OCS-JA, CAS-JA and SCAS-GAD, CAS-JA and K6, OCS-JA and SCAS-OCD, and OCS-JA and K6 were 0.805, 0.371, 0.322, 0.658, and 0.472, respectively. The threshold for discriminant validity by HTMT was less than 0.85 (Henseler, Ringle & Sarstedt, 2015); thus, the CAS-JA and OCS-JA met the criterion for discriminant validity.

### Convergent validity and inter-item correlations

Inter-item correlations of the CAS-JA and OCS-JA are shown in Tables 2 and 3, respectively. The composite reliabilities were 0.875 (CAS-JA) and 0.834 (OCS-JA). AVE was 0.540 (CAS-JA) and 0.609 (OCS-JA). The threshold for AVE is 0.50 (Fornell & Larcker, 1981), and these values were higher than the threshold.

### Differences between sociodemographic groups

No differences in the total scores for the CAS-JA and OCS-JA were found in the WRS test. Comparing the state of emergency and non-emergency areas, their total CAS-JA scores were 0.978 and 0.897, respectively (*p* = 0.436). The values of the OCS-JA were 1.562 and 1.259 (*p* = 0.146) respectively. Similarly, based on gender, no differences were found in the total scores of the CAS-JA and OCS-JA. The CAS-JA values for men and women were 1.226 and 0.831 (*p* = 0.358), respectively, and the OCS-JA values were 1.452 and 1.333 (*p* = 0.710), respectively.

In the CFA, the CAS-JA and OCS-JA had no statistically significant relationship with either the state of the area (*i.e.,* emergency or non-emergency) or gender. The effect of the former on the CAS-JA was 0.011 (*p* = 0.176), and on the OCS-JA, it was 0.007 (*p* = 0.320); that of the latter on the CAS-JA was −0.144 (*p* = 0.436), and on the OCS-JA, it was −0.093 (*p* = 0.595).

## Discussion

Previous studies have suggested that the CAS and OCS have single-factor structures (Lee, 2020a; Magano et al., 2021; Ahmed et al., 2022; Ashraf, Lee & Elizabeth Crunk, 2022; Broche-Pérez et al., 2022; Choi, Lee & Lee, 2022; Evren et al., 2022). In this study, the single-factor models of the CAS-JA and OCS-JA were found to fit well, for all indices. The threshold for test–retest reliability by ICC was proposed to be above 0.75, which was met in the CAS-JA and OCS-JA.

The CAS-JA and SCAS-GAD both measured anxiety levels, and the correlation was 0.328 (*p* < 0.001). Although this value was statistically significant, it was not high. Additionally, we calculated the HTMT to determine discriminant validity and confirmed that each scale was discriminant. The results indicated that both scales measure anxiety but focus on various aspects of anxiety. Moreover, the SCAS-GAD, which measures the symptoms of generalized anxiety disorder, cannot measure COVID-19-related anxiety. The same applies to the OCS-JA and SCAS-OCD.

The inter-item correlations for the CAS-JA ranged from 0.504 −0.655, whereas that for the OCS-JA ranged from 0.377 −0.668. All items had moderate to high correlation coefficients for the CAS-JA and OCS-JA. These results show that each item on the CAS-JA and OCS-JA measured similar constructs, but not entirely similar constructs either. We also checked the convergent validity to calculate composite reliability and AVE. Because all the values were above the thresholds, the convergent validity was adequate for the CAS-JA and OCS-JA.

There were no gender differences in the original CAS English version (Lee, 2020a). In the CAS Turkish version (Evren et al., 2022) and CAS for Mexican healthcare workers (Mora-Magaña et al., 2022), the scores of women were higher than those of men. By contrast, in the OCS Urdu version (Ashraf, Lee & Elizabeth Crunk, 2022), the scores of men were higher than those of women. In this study, no differences were found among sociodemographic groups in either the WRS test or CFA. In these previous studies, the gender differences for the CAS and OCS were not consistent. This result could be due to the sampling method, language characteristics, country, and infection status.

This study has several limitations. First, only high school students were surveyed, and younger teenagers were not included. Second, our survey used an online sample. Therefore, it may not be completely representative of Japanese high school students. The survey was conducted during the COVID-19 pandemic, and online implementation was appropriate from the perspective of infection prevention.

In this study, we created the CAS-JA and OCS-JA questionnaires and assessed their factor structure, internal consistency reliability, test–retest reliability, discriminant validity and inter-scale correlations, convergent validity and inter-item correlations, and differences between sociodemographic groups.

Given that both the CAS-JA and the OCS-JA are short (5 and 4 items, respectively), they are suitable for social surveys in sociology and social psychology. Moreover, our results indicate that general anxiety/obsession differs from anxiety/obsession about COVID-19. As such, these scales would provide a more sensitive measurement of anxiety/obsession regarding COVID-19. Another advantage is that the 4–5-item questionnaires can easily fit the screen of a smartphone, facilitating the delivery of these psychometric tools.

In Japan, the mortality rate from COVID-19 is low as a result of vaccination progress (Dong, Du & Gardner, 2020; Johns Hopkins University of Medicine, 2021). However, some students potentially have a high level of anxiety and obsession with COVID-19. Without the proper tools, it is impossible to assess the status of these students. The Japanese versions of the CAS-JA and OCS-JA developed here, which can measure adolescents’ anxiety and obsession with COVID-19, could help facilitate screening and thereby promote medical examinations and treatment related to anxiety/obsession about COVID-19. Japanese schools were suspended from March to May 2020 but have subsequently provided in-person classes. Although the Ministry of Education, Culture, Sports, Science and Technology (MEXT) has noted students’ refusal to go to school because of COVID-19 (MEXT, 2021), no detailed quantitative analysis of anxiety or obsession with COVID-19 has been conducted. It is expected that the CAS-JA and OCS-JA developed here would be useful in assessing the actual status of such students.

## Conclusions

This study examined psychometric properties, such as reliability and validity, of the CAS-JA and OCS-JA questionnaires for the measurement of COVID-19-related anxiety, and persistent and disturbed thoughts (also known as obsessions) among Japanese adolescents. The CFA results validated the single-factor structure of these questionnaires. Our findings also showed good test–retest reliability, internal consistency, discriminant validity, and convergent validity. In short, the CAS-JA and OCS-JA proved to be valid questionnaires for measuring COVID-19-related anxiety, and persistent and disturbed thoughts in Japanese adolescents. These questionnaires can help identify mental health issues in Japanese adolescents and aid in their early diagnosis and treatment. The CAS-JA and OCS-JA were originally designed for Japanese junior high school students (12 years or older). Although this study of the scale characteristics was conducted on high school students owing to technical problems, the content of the scales is simple enough to be understood by junior high school students. As anxiety and obsession tend to begin during adolescence and subsequently become chronic, scales that can measure anxiety and obsession related to COVID-19 would be of great use.

##  Supplemental Information

10.7717/peerj.15710/supp-1 Supplemental Information 1 Coronavirus Anxiety Scale (CAS) questionnaire (in Japanese)

10.7717/peerj.15710/supp-2 Supplemental Information 2 Obsession with COVID-19 Scale (OCS) questionnaire (in Japanese)

## Additional Information and Declarations

### Data Availability

The following information was supplied regarding data availability: The datasets presented in this article are not readily available because consent for data disclosure was not obtained at the time consent was obtained. Requests to access the datasets may be directed to the Division for Research Promotion for Matsuoka Campus Research and Regional Cooperation Promotion Department University of Fukui (rinsho-rinri@ml.u-fukui.ac.jp).
