# Supplementary material for: Validity and reliability of the Japanese versions of the coronavirus anxiety scale for adolescents and obsession with COVID-19 scale for adolescents
Source: PeerJ. 2023 Aug 9;11:e15710. doi: 10.7717/peerj.15710 (PMC10422950; doi:10.7717/peerj.15710)
Supplement: Supplemental Information 1 [file peerj-11-15710-s001.docx]

| **CAS-JA** | | | | | | | |
| --- | --- | --- | --- | --- | --- | --- | --- |
|  |  |  |  |  |  |  |  |
| この2週間に次のようなことをどれくらい経験しましたか。 | |  | 全くない | まれ  1-2日未満 | 数日 | 7日以上 | この2週間ほぼ毎日 |
|  |  |  |  |  |  |  |  |
| 1. | コロナウイルスのニュースを読んだり聞いたりしたとき、めまいがしたり、頭がふらふらしたり、気が遠くなるような感じがした |  | 0 | 1 | 2 | 3 | 4 |
| 2. | コロナウイルスのことを考えていて、なかなか寝られなかったり、しっかり寝られなくなったりした |  | 0 | 1 | 2 | 3 | 4 |
| 3. | コロナウイルスのことを考えたり、見たり聞いたりすると、マヒしたり、固まってしまったりするような感じがした |  | 0 | 1 | 2 | 3 | 4 |
| 4. | コロナウイルスのことを考えたり、見たり聞いたりすると、食欲がなくなった |  | 0 | 1 | 2 | 3 | 4 |
| 5. | コロナウイルスのことを考えたり、見たり聞いたりすると、吐き気がしたり、おなかの調子が悪くなったりした |  | 0 | 1 | 2 | 3 | 4 |
|  |  |  |  |  |  |  |  |
|  | コラム合計 |  | + | + | + | + | + |
|  |  |  |  |  |  |  |  |
|  |  |  |  |  | 総得点 | | |
|  |  |  |  |  |  |  |  |
