# Supplementary material for: Validity and reliability of the Japanese versions of the coronavirus anxiety scale for adolescents and obsession with COVID-19 scale for adolescents
Source: PeerJ. 2023 Aug 9;11:e15710. doi: 10.7717/peerj.15710 (PMC10422950; doi:10.7717/peerj.15710)
Supplement: Supplemental Information 2 [file peerj-11-15710-s002.docx]

| **OCS-JA** | | | | | | | |
| --- | --- | --- | --- | --- | --- | --- | --- |
|  | | | | | | | |
| この2週間に次のようなことをどれくらい経験しましたか。 | |  | 全くない | まれ  1-2日未満 | 数日 | 7日以上 | この2週間ほぼ毎日 |
|  |  |  |  |  |  |  |  |
| 1. | コロナウイルスにかかってしまったのではないかという考えに悩まされた |  | 0 | 1 | 2 | 3 | 4 |
| 2. | コロナウイルスにかかった人と会ったかもしれないという考えに悩まされた |  | 0 | 1 | 2 | 3 | 4 |
| 3. | コロナウイルスについて考えることをやめられなかった |  | 0 | 1 | 2 | 3 | 4 |
| 4. | コロナウイルスについての夢を見た |  | 0 | 1 | 2 | 3 | 4 |
|  |  |  |  |  |  |  |  |
|  | コラム合計 |  | + | + | + | + | + |
|  | |  |  |  |  |  |  |
|  |  | 総得点 | | | | | |
